# *Ngaa-bi-nya-nhumi-nya* (to Test First): Piloting the Feasibility of Using the Growth and Empowerment Measure with Aboriginal Pregnant Women Who Smoke

**DOI:** 10.1155/2021/6610500

**Published:** 2021-01-13

**Authors:** Michelle Bovill, Yael Bar-Zeev, Billie Bonevski, Jennifer Reath, Christopher Oldmeadow, Alix Hall, I. C. A. N. Q. U. I. T. in Pregnancy Pilot Group, Gillian S. Gould

**Affiliations:** ^1^The University of Newcastle, Callaghan, New South Wales, Australia; ^2^Hunter Medical Research Institute, New Lambton, New South Wales, Australia; ^3^Western Sydney University, Penrith, New South Wales, Australia

## Abstract

**Introduction:**

Aboriginal pregnant women who smoke experience barriers to quitting, including challenges to social and emotional well-being, but these are infrequently quantified. Finding an appropriate measurement tool in this setting is crucial to increase knowledge for holistic smoking cessation interventions.

**Aims:**

To pilot the Growth and Empowerment Measure (GEM) with a sample of pregnant Aboriginal women who smoke.

**Methods:**

Aboriginal women participating in the step-wedge ICAN QUIT in Pregnancy pilot study completed the GEM comprised of 14-item Emotional Empowerment Scale (EES14), 12 Scenarios (12S), and K6 items at baseline, 4 weeks, and 12 weeks. Qualitative interviews with service staff were held at the end of the study to assess feasibility.

**Results:**

15 pregnant Aboriginal women took part between November 2016 and July 2017. At 12 weeks, *n* = 8/12 (67%) of women reported an increase in both the EES14 and 12S scores. Total 12S scores were significantly higher at 12 weeks (*p* = 0.0186). Total K6 had a nonsignificant trend for reduction (*p* = 0.0547). Staff reported that the length of the survey presents challenges in this setting.

**Conclusions:**

A shortened, modified GEM is recommended in this setting. We recommend the GEM to be tested in a larger study, powered to assess its associations with smoking behaviours.

## 1. Introduction

Aboriginal people define health not only as a state of physical well-being but rather a holistic concept which incorporates social, emotional, and cultural well-being [1]. Aboriginal academics have called for initiatives that empower Aboriginal people, to regain control of their lives and in turn enhance social and emotional well-being (SEWB) [2, 3]. The National Aboriginal and Torres Strait Islander Health Plan 2013-2023 states “Aboriginal and Torres Strait Islander peoples have the right to live a healthy, safe and empowered lives with a healthy strong connection to culture and country” [4]. This plan also prioritises SEWB as a central platform for preventive and clinical care [4]. Culture and empowerment underpin the priorities set by the Australian Government to improve Aboriginal health and well-being, yet empowerment is seldomly reported as an outcome measure for health interventions. The term empowerment is complex and subjective, and its use varies widely depending on context. Empowerment approaches should be at the forefront of ethical Aboriginal health research acknowledging colonisation, imperialism, dispossession, and systemic racism that have impacted Aboriginal culture, and as a direct result, health and well-being. Empowerment is a strength-based approach that identifies capabilities rather than cataloguing risk factors and reporting deficits [5]; as such, it can be understood as reempowerment [3]. It is through a reempowerment that Aboriginal people can “achieve their full potential as a human being, thereby bringing about the total wellbeing of their community” which is an Aboriginal definition of health [4]. Empowerment research values the process in which individuals and communities gain greater access and control of their lives [6]. Empowerment approaches for Aboriginal people are based on the same fundamental principles as the general population; however, they must also address personal values and the development of skills for helping others [7].

Health inequities between Aboriginal and non-Aboriginal Australians are monitored and reported under the “Closing the Gap” campaign. Reducing smoking during pregnancy is a focus for meeting the “Closing the Gap” target of halving the gap in mortality rates for Aboriginal children under five within a decade [4, 8]. Aboriginal pregnant women are more than three times as likely to smoke during pregnancy than their non-Aboriginal counterparts (43% vs. 12%) [9] with the relative gap in prevalence widening over time [10]. Aboriginal people are also nearly three times more likely to experience high or very high psychological distress than non-Aboriginal people, with Aboriginal women experiencing higher rates of distress than Aboriginal men across all age groups apart from 45-54 years [11]. The association between smoking and psychological distress among Aboriginal people is starting to be explored [12, 13]. Psychological distress in pregnancy is independently associated with babies born with low birth weight [14], as is maternal smoking [15].

Aboriginal women have reported factors that may influence continued smoking during pregnancy such as experiencing high levels of stress and trauma [16] and wanting to maintain social environments and relationships by smoking [17]. While stress and trauma are acknowledged to influence continued smoking during pregnancy [16], this has been seldomly explored in association with quitting. Given the complex intergenerational consequences of colonisation, dispossession, and removal of Aboriginal children affecting the social and emotional well-being (SEWB) of Aboriginal people today [18], it is important to use culturally responsive tools to measure SEWB within smoking cessation interventions. With increased knowledge of the association between SEWB and successful quitting, interventions can be tailored to enhance quitting success among pregnant Aboriginal women. Approaches to encourage and empower Aboriginal women have been recommended to reduce the prevalence in smoking during pregnancy [19]. In order to explore these approaches, it is important that empowerment outcome measures be incorporated within interventions in this setting.

As far as we are aware, the GEM has not been used previously among pregnant women, or for smoking cessation research. This study is aimed at piloting the use of the GEM with a sample of pregnant Aboriginal women who smoke and explore the feasibility of this tool in the Aboriginal Community Controlled maternal healthcare and smoking cessation setting.

## 2. Methods

This was a nested study within the step-wedge ICAN QUIT in Pregnancy pilot study. The ICAN QUIT in Pregnancy pilot is aimed at enhancing health provider provision of smoking cessation support offered to Aboriginal women, or women who were pregnant with an Aboriginal baby, attending Aboriginal Community Controlled Health Services (ACCHS). The ICAN QUIT in Pregnancy intervention offered webinar training, educational resources, and free oral nicotine replacement therapy (NRT) to ACCHS. The study protocol [20], development process [19, 21], and results [22–24] have been reported elsewhere. The study was developed using the Behaviour Change Wheel, incorporating the COM-B (capability, opportunity, and motivation for behavioural interventions) and a Theoretical Domains Framework [25]. Six Aboriginal Community Controlled Health Services in NSW, SA, and Qld participated in the small cluster step-wedge pilot trial with health providers receiving webinar training, educational resources, and free oral NRT in three clusters, two months apart. Research facilitators were recruited and trained in each ACCHS by the research team to conduct the study on site. Research facilitators were trained health providers in the ACCHS; some sites had two staff as research facilitators due to workload planning. Research facilitators recruited women and conducted the GEM survey data collection with the participants.

There is only one evaluative tool to measure empowerment has been designed with/for and validated by Aboriginal people. The Growth and Empowerment Measure (GEM) is a psychometrically validated tool that was developed and tested in partnership with Aboriginal people [26]. Psychometric property analysis had included interitem correlations, relationships between subscales, and a principle component analysis. The GEM measures change in dimensions of empowerment as defined and described by Aboriginal people [26]. This tool addresses the influence of SEWB on family, organisational and community levels, thus connecting with the Aboriginal health definition. The GEM Scale also includes the Kessler 6 Psychological Distress Scale (K6) measure of psychological distress. The GEM was first used with the Family Wellbeing Program. This program reported the effectiveness of empowerment-based approaches and measures to facilitate people's capacity to regain social and emotional well-being (SEWB) and begin to rebuild the social norms of their families and community [27–29]. The GEM is a relatively new tool that has only been reported in three other studies: the evaluation of an arts-based program [30], a residential rehabilitation substance abuse centre [31], and with child protection staff in five remote North Queensland indigenous communities [27].

### 2.1. Participants

#### 2.1.1. Aboriginal Women

Eligibility criteria comprised Aboriginal women, ≤28-week gestation, aged 16 years old or more, and who smoked tobacco at any level of consumption. Women were not required to quit smoking or to intend to quit as part of entry into the study. Participants were recruited between November 2016 and July 2017 with a follow-up completed by September 2017. Aboriginal women recruited to the study were deidentified, as such the research team could not approach any women to discuss the feasibility of the survey tool. Consultation with the Aboriginal Advisory Group occurred to seek alternative ways to understand Aboriginal women's views on the study. Directly approaching women who were heavily pregnant or who had recently given birth to seek their views was not deemed appropriate by the advisory group.

#### 2.1.2. Service Staff

At the end of the study, all service staff with an involvement in the study were invited to participate in qualitative interviews to assess the feasibility of the ICAN QUIT in Pregnancy pilot study as a whole. Interviews were conducted between August 2017 and January 2018 by MB and GG either face to face at the service on over telephone (for staff unavailable at the time of visiting the service).

Ethics is discussed as follows: the study was developed in collaboration and negotiation with Aboriginal communities in New South Wales; this process was reported previously [19]. This study was also overseen by an Aboriginal Advisory Group made up of staff from each partnering Aboriginal Community Controlled Health Service to ensure that the research design and implementation was culturally safe and appropriate for their community. The study was approved by the University of Newcastle Human Research Ethics Committee (HREC) (#H-2015-0438), AH&MRC HREC (#1140/15), and AHREC HREC (#04-16-652).

#### 2.1.3. Procedure

As part of the ICAN QUIT in Pregnancy pilot study, women completed three study visits at recruitment, 4-week and 12-week follow-up. Women were offered $20 shopping vouchers for each visit, as a reimbursement for their time.

#### 2.1.4. Survey Instrument

Women completed two surveys (20 minutes in total) at each study visit, administered by a qualified health provider at the ACCHS who received training by the study team to perform the role of research facilitator. Training included how to gain informed consent and administer the following surveys:

(1) Demographic and smoking characteristic survey included assessing the participant's age, Aboriginal status, partner status, number of children, smoking status, number of cigarettes smoked per day, number of quit attempts, and length of cessation
(2) GEM and K6: the GEM included two empowerment scales and the K6 psychological distress scale:
  - The *14-item Emotional Empowerment Scale (EES14)* asked women to indicate, on a five-point Likert scale, how they felt about themselves. The EES14 comprised of two subscales. Inner Peace subscale included questions on feeling skillful, strong and full of energy, happy with self and life, confidence, centred and focused, calm and relaxed, safe and secure, and dealing with anger. Self-Capacity subscale included questions on satisfaction with opportunities, feeling valued and admired, speaking out and people listening, belonging and connection, and hope for a better future
  - *12 Scenarios (12S)* asked women to indicate, using a seven-point Likert scale, how they viewed themselves in relation to each of 12 scenarios. The 12S consists of two subscales. Healing and Growth subscale covered scenarios relating to dealing with painful feelings, personal and family safety, being able to say no, engaging with learning, ability to speak out and be heard, reaction to judgment, and improving relationships. Connection and Purpose subscale covered scenarios related to ability to make changes, recognising one's own spirituality, sense of identity, being valued in the workplace, and working toward a better community. The workplace questions were omitted in this study in consultation with Aboriginal communities as it was deemed not to be relevant for Aboriginal women during pregnancy and could cause shame; omitting these would not impact analysis as measures were asked as changes over time). The GEM subscores were calculated by summing the relevant items together and dividing by the number of items in the specific scale. For psychometrics, properties of this scale refer to psychometric validation paper by developers Haswell et al. [26]
(3) *K6 (psychological distress scale)* is a 6-item questionnaire measuring self-reported mood and anxiety disorders on a 5-point Likert scale (1 = “none of the time” to 5 = “all of the time”). Women were asked to rate how they felt in the last 4 weeks, for each of the following items: (i) *nervous*, (ii) *hopeless*, (iii) *restless or fidgety*, (iv) *so depressed that nothing could cheer you up*, (v) *that everything was an effort*, and (vi) *worthless.* The K6 was scored using the Australian scoring system. Possible scores ranged from 6 to 30, dichotomised to low (score 6-14) and high distress (score 15-30) based on recommended cut points [32]

#### 2.1.5. Qualitative Interviews

Semistructured interviews were conducted by MB and GG with service staff (including managers, health professionals, and research facilitators) face to face or over the phone to assess acceptability of the intervention. No direct questions regarding the GEM survey tool were included; however, general questions on the challenging aspects of the study provided data relevant to this paper. Interviews were conducted using open-ended questions; overall feasibility and acceptability of the ICAN QUIT in Pregnancy study was addressed which incorporated the implementation of survey tools. Interviewers asked “I am interested to learn about the implementation of ICAN QUIT in Pregnancy at your service. Please feel free to share your observations.”

#### 2.1.6. Data Analysis

Statistical analyses were programmed using SAS v9.4 (SAS Institute, Cary, North Carolina, USA). Descriptive statistics, including means, standard deviations, medians, and interquartile ranges, are presented for the main outcomes for each of the three time periods.

EES14, 12S, and K6 scores were compared for participant's baseline and 12-week surveys. A paired sample *t*-test was used to compare participants' mean EES14 scores, while the Wilcoxon signed-rank test was used to compare women's median total scenario score and K6 score, as the data for these outcomes violated the assumption of normality. Only women who had data from both baseline and the 12-week follow-up were included in these comparisons.

Qualitative interviews were audio-recorded and professionally transcribed. Transcripts were thematically analysed [33]. Data reflecting feasibility of the GEM survey are reported here. Data were analysed using NVivo 11 software by two independent coders.

## 3. Results

Four out of the six ACCHS piloted the GEM survey; one site did not recruit eligible women to the study and another site reported being unable to complete the survey with participants. Two women were excluded from the analysis as they were not Aboriginal (women were mothers of Aboriginal babies) and this pilot is aimed at assessing feasibility for Aboriginal pregnant women who smoke. Fifteen Aboriginal women participated from the four ACCHS in NSW and SA. Fifteen Aboriginal women completed the GEM at baseline, *n* = 13 at the four-week follow-up, and *n* = 12 at the 12-week follow-up. Inspection of the GEM and K6 scores did not show any significant trends between women who dropped out compared to those who remained in the study.

As shown in Table 1, the majority of participants identified as Aboriginal and had already given birth to a previous child. Almost three quarters had a partner and just over half smoked every day. The average age of participants was 27 years, and the average duration of pregnancy in weeks was 19.2.

### 3.1. GEM

Table 2 reports the mean scores for the EES14, 12S, and K6 at each time point for all women.

The differences between women's baseline and 12-week EES14 and K6 scores were not significant (Table 3). However, the difference between women's baseline and 12-week 12S scores was significant, with median scores higher at the 12-week follow-up (Table 3).

### 3.2. K6

Nine of the 15 women (60%) at baseline and 7 of 13 women (54%) at the 4-week follow-up were classified as having high distress. Only 4 of 12 women (33%) had high distress at the 12-week follow-up. Of the nine participants who had high distress at baseline, only one reduced their distress at the 4-week follow-up. However, 50% of participants who reported high distress at baseline subsequently reported low distress at the 12-week follow-up. One participant went from low distress at baseline to high distress at the 4-week follow-up.

### 3.3. GEM Feasibility and Useability

Eighteen service staff participated in qualitative interviews across all six ACCHS. Four research facilitators in three services made comments about the GEM survey. One site reported being unable to implement the GEM survey in data collection due to the long timeframe to complete the survey. Site locations have not been added to reporting results to maintain anonymity of staff.

“Because - just timing basically. Yeah, because my appointments go for an hour. So - and in those appointments I do give a lot of antenatal education. We talk about what they've missed, what they need to have, in terms of antenatal care.”—Research Facilitator.

While the research facilitator did ask women to complete the survey, due to time constraints and multiple competing demands in an Aboriginal Community Controlled healthcare setting, the survey was not completed.

“I didn't have time to go through survey forms with patients. So I just left that to them.”—Research Facilitator.

Another service that was able to collect the GEM surveys also reported challenges in implementing such a long survey in their Aboriginal Community Controlled healthcare setting.

“I really had to just remind them to expect that because, like, it's so long and it's interesting that because that's made for Aboriginal people… there was a couple of clients where I had to read out the questions for them because they didn't want to read them…. it's a really long survey even I didn't like reading it out.”—Research Facilitator.

While one staff member commented on the intensity of the questions asked in the survey, it was acknowledged that it was to be expected from a social and emotional well-being survey.

“It's the heavy questions and that, so just take your time.' …I really had to just remind them to expect that… anything to do with social and emotional stuff is going to be challenging, especially… if you had a bad week.”—Research Facilitator.

## 4. Discussion

This ICAN QUIT in Pregnancy study piloted the use of the GEM for the first time with 15 pregnant Aboriginal women, measuring SEWB during pregnancy and assessed feasibility with service staff. To our knowledge, this is the first application of the GEM in smoking or pregnancy, offering a pilot use of the GEM within this setting. Total scenario scores (*healing and enabling growth and connection and purpose*) significantly increased over time. The scenarios measure functional aspects of empowerment and were derived from qualitative interviews with Aboriginal people [26]. Questions such as “How do you feel about making changes in your life?” are asked in the scenarios and as such offer an important positivist outcome measurement. In general, women's psychological distress appeared to reduce over time, with 50% of participants who reported high distress at baseline subsequently reporting low distress at the 12-week follow-up; although this result is not clinically significant, this result is promising and could be measured with the K6 in a larger sample. If we acknowledge that empowerment can lead to long-term enhancement of Aboriginal health and well-being [4], reporting a significant change in functional aspects of empowerment by Aboriginal women may signal long-term success in smoking cessation, as multiple opportunities for intervening have been previously reported [34].

Aboriginal women have reported high levels of stress and trauma [16], wanting to maintain social environments and relationships [17], a desire to maintain ownership of the quitting process [19], and a need for enhanced knowledge and resources to achieve cessation [35]. The 12S scenarios seem to address these stated needs and additional areas of empowerment important to Aboriginal people including spirituality, a sense of identity, and working toward having a better community [7]. The influence of stress and trauma [16] may not be appropriately measured using the K6 for pregnant Aboriginal women. This is possibly due to the complex intergenerational effects of colonisation, dispossession, and removal of Aboriginal children effecting SEWB, as well as experiences of social and economic disadvantage [18] not being specifically addressed in this questionnaire. As stress and trauma are primary influences on Aboriginal women smoking during pregnancy, it is important to enhance data collection during pregnancy to better understand how interventions can target these areas.

The GEM is a relatively new measurement tool for Aboriginal SEWB and has only been reported in four other studies: the evaluation of an arts-based program [30], a residential rehabilitation substance abuse centre [31], with child protection staff in five remote North Queensland indigenous communities [27] and postgraduate students participating in an Aboriginal Public Health Perspectives course [36]. Participants in the rehabilitation substance abuse centre reported a significant decrease in psychological distress and an increase in empowerment over 8 weeks [31]. These findings indicate the GEM is a useful measurement tool for an addiction setting; however, applying this to pregnancy and smoking will require further assessment. Our study found one subscale to change significantly over time with potential for other significant trends if applied to a larger sample. Empowerment research is strength-based, identifying capabilities rather than cataloguing risk factors and reporting deficits [5]. Identifying appropriate supports for Aboriginal women during pregnancy may lie in positivist reporting and ensuring interventions build on particular capabilities.

While qualitative interviews with staff did not find any concerns with the questions asked in the GEM being acceptable for Aboriginal women in a maternal or smoking cessation intervention, the length of the survey is problematic in this setting. Only four of the six sites in the ICAN QUIT in Pregnancy study participated in GEM survey; this was due to one site not recruiting any eligible Aboriginal mothers and the other site reporting the survey tool was too long to implement. Conducting research with Aboriginal women during pregnancy has been reported to be challenging [37]. Refining assessment tools that connected with Aboriginal definition of health and well-being is recommended to enhance Aboriginal statistical reporting and move away from purely deficit reporting [38]. While GEM is an appropriate and acceptable tool for measuring SEWB with Aboriginal people [26, 28], it may be too long for pregnant women to give other health and medical appointments and regular follow-ups. A shortened version of the GEM has been developed and trialed which reduces the 12S to a core 6-item questionnaire [36]; this should be used in further exploration using the GEM.

## 5. Strengths and Limitations

This is the first study to address the feasibility of the GEM survey in an Aboriginal Community Controlled maternal health setting and a smoking cessation setting. This study was conducted with a small sample of Aboriginal women participating in a smoking cessation study. The study originally sought to explore the use of the GEM to measure possible associations between smoking behaviours and SEWB. Due to a lower rate of recruitment than anticipated, this was not able to be achieved. The feasibility of the GEM tool in this setting was only assessed by service providers which do present a limitation; however, conducting respectful and ethical research with Aboriginal people must also respect community priorities and not overburden participants. It was the view of service staff who participated on the Aboriginal Advisory Group that contacting women for interviews was not appropriate due to them being heavily pregnant or having recently given birth. Women also expressed to staff that they did not want to be approached by researchers. Due to the close and long-term relationships between ACCHS staff and participants, the research team deemed it valid to assess feasibility of the tool with service staff.

This study has reported feasibility data that is useful and informative to further applications of the GEM in these settings.

## 6. Conclusion

Empowerment is frequently referenced in relation to Aboriginal health, yet seldom used as a measurement within interventions to improve Aboriginal health. This study piloted the GEM with 15 pregnant Aboriginal women during pregnancy from NSW and SA, identifying changes in empowerment over time. It is important to measure health interventions in line with priorities within the National Aboriginal and Torres Strait Islander Health Plan, and our findings suggest the GEM may be an appropriate tool to do this; however, the survey length is a challenge in this setting. The shortened version of the tool (core 6) is recommended for use in Aboriginal primary care. These findings warrant further investigation in a larger trial of Aboriginal smoking cessation during pregnancy, and we recommend also seeking feedback from Aboriginal participants.

## Figures and Tables

**Table 1:** Baseline characteristics of participants.

	*n*	**%**

Identify as Aboriginal		
No	1	6.7
Yes	14	93.3

Identify as Torres Strait Islander		
No	14	93.3
Yes	1	6.7

Identify as Aboriginal and Torres Strait Islander		
No	12	80.0
Yes	3	20.0

Partner		
No	4	26.7
Yes	11	73.3

Number of children given birth to		
None	1	6.7
1 to 2	7	46.7
3 or more	7	46.7

Education		
Primary or up to year 9	5	33.3
Year 10 to 11	7	46.7
Year 12	2	13.3
TAFE/currently at university	1	6.7

Smoking status		
Everyday	8	53.3
Most days	4	26.7
Occasionally	3	20.0

	Mean	SD

Age	27.2	5.5
Duration in pregnancy	19.2	8.5

**Table 2:** Descriptive statistics for the main variables of interest, in Aboriginal women participating in ICAN QUIT in Pregnancy study.

Comparison group	Baseline mean (SD)	Baseline median (IQ1, IQ3)	4-week follow-up mean (SD)	4-week follow-up median (IQ1, IQ3)	12-week follow-up mean (SD)	12-week follow-up median (IQ1, IQ3)
EES14	3.51 (0.70)	3.50 (2.93, 4.00)	3.59 (0.85)	3.79 (2.86, 4.21)	3.81 (0.73)	3.61 (3.14, 4.50)
12S	4.07 (0.98)	4.18 (3.55, 4.64)	4.57 (1.43)	4.73 (3.36, 5.27)	4.88 (1.12)	4.82 (4.09, 5.00)
K6 score	14.40 (4.05)	15.00 (10.00, 18.00)	14.00 (4.14)	16.00 (11.00, 17.00)	13.17 (3.33)	14.00 (10.00, 15.50)

**Table 3:** Comparisons of 12-week and baseline scores for EES14, 12S, and K6 scores, in Aboriginal women participating in ICAN QUIT in Pregnancy study (*n* = 12).

Comparison group	Baseline mean (SD)	12-week follow-up mean (SD)	Baseline median (IQ1, IQ3)	12-week follow-up median (IQ1, IQ3)	*p* value
12-week EES14 vs. baseline EES14	3.55 (0.76)	3.81 (0.73)	3.61 (2.86, 4.07)	3.61 (3.14, 4.50)	0.2982^a^
12-week 12S vs. baseline 12S score	4.20 (1.00)	4.88 (1.12)	4.32 (3.59, 4.64)	4.82 (4.09, 5.00)	0.0186^b^
12-week K6 score vs. baseline K6 score	14.67 (4.19)	13.17 (3.33)	15.50 (11.00, 18.00)	14.00 (10.00, 15.50)	0.0547^b^

^a^Paired sample *t*-test. ^b^Wilcoxon signed-rank test.

## Data Availability

No data is available.
